# Efficacy and safety of intravenous tranexamic acid administration in patients undergoing hip fracture surgery for hemostasis: A meta-analysis

**DOI:** 10.1097/MD.0000000000006940

**Published:** 2017-05-26

**Authors:** Pei Zhang, Jinshan He, Yongchao Fang, Pengtao Chen, Yuan Liang, Jingcheng Wang

**Affiliations:** a Department of Orthopedics, Clinical Medical College of Yangzhou University, Subei People's Hospital, Yangzhou, China; b Dalian Medical University, Dalian, Liaoning, China.

**Keywords:** hip fracture, meta-analysis, surgery, tranexamic acid

## Abstract

**Background:**

Patients undergoing hip fracture surgery frequently require blood transfusion. Tranexamic acid (TXA) has been widely used to decrease blood loss and transfusion rates in joint replacement surgery. Therefore, we conducted a meta-analysis to evaluate efficacy and safety of intravenous TXA administration in patients suffering from hip fractures.

**Methods:**

Electronic databases were searched before December 2016 by 2 independent reviewers, including Cochrane Library, EMBASE, PubMed, Web of Science, the Chinese Biomedical Literature database, and the China National Knowledge Infrastructure databases. Randomized controlled trials (RCTs) involving the efficacy and safety of intravenous (IV) TXA in patients who underwent hip surgery were included in our meta-analysis. The endpoints included total blood loss, hidden blood loss, postoperative hemoglobin decline, transfusion rates, the rate of thrombotic events, and operative time. Current meta-analysis was performed following the guidelines of the Cochrane Reviewer's Handbook and the PRISMA statement. The pooling of data was carried out using STATA V.12.0 software.

**Result:**

Eight RCTs were included, involving 598 participants. Current meta-analysis indicated that the IV TXA group had less total blood loss (weighted mean difference [WMD] = −277, 95%CI: −335 to −220, *P* = .000), less hidden blood loss (WMD = −246, 95%CI: −252 to −241, *P* = .000), lower postoperative hemoglobin decline (WMD = −1.36, 95% CI: −1.84 to −0.88, *P* = .000), and lower transfusion rates (risk difference [RD] = −0.19, 95% CI: −0.27 to −0.11, *P* = .000) compared to the control group. No significant differences were found regarding the rate of thrombotic events (RD = 0.02, 95% CI: = −0.01 to 0.05, *P* = .262) and operative time (WMD = −0.7, 95% CI: −3.3 to 1.9, *P* = .6).

**Conclusion:**

It was well established that systemic administration of TXA could reduce blood loss and transfusion rates in hip fracture surgery. But the optimal regimen, dosage, and timing still need a further research. In addition, more large and high-quality randomized controlled studies are needed to focus on the safety of IV TXA application before its wide recommendation for use in hip fracture surgery.

## 1 Introduction

With the aging of society and the increasing number of old people with osteoporosis, the incidence rate of hip fractures is increasing.^[1]^ Hip fracture as a common type of fractures frequently results in considerable blood loss,^[2]^ exposing patients to postoperative anemia which could lead to a reduced functional recovery and a detrimental effect on long-term mortality.^[3]^ Blood transfusion could correct anemia. However, blood transfusion is associated with increased incidence rates of adverse effects, such as infectious diseases, hemolytic reaction, cardiovascular dysfunction, and postoperative infection.^[4–7]^ Therefore, to traumatic orthopedists, it is an important issue to reduce perioperative blood loss during the treatment of hip fractures.

Tranexamic acid (TXA) as a type of synthetic amino acid analog could block the lysine-binding sites on plasminogen to inhibit the activation of plasminogen and finally interfere with fibrinolysis.^[8]^ Now, TXA is widely used in urological, gynecological, and thoracic surgery,^[9–12]^ and numerous previous studies have proved that intravenous (IV) TXA could decrease blood loss and transfusion rates without increasing thrombotic events in joint arthroplasty.^[13–15]^ However, there is limited data on the efficacy and safety of IV TXA in hip fractures, and whether IV TXA should be used in hip fracture remains controversial. So, we performed this meta-analysis to investigate the efficacy and safety of IV TXA in patients suffering from hip fractures.

## 2 Materials and methods

### 2.1 Literature search

**Figure 1:**
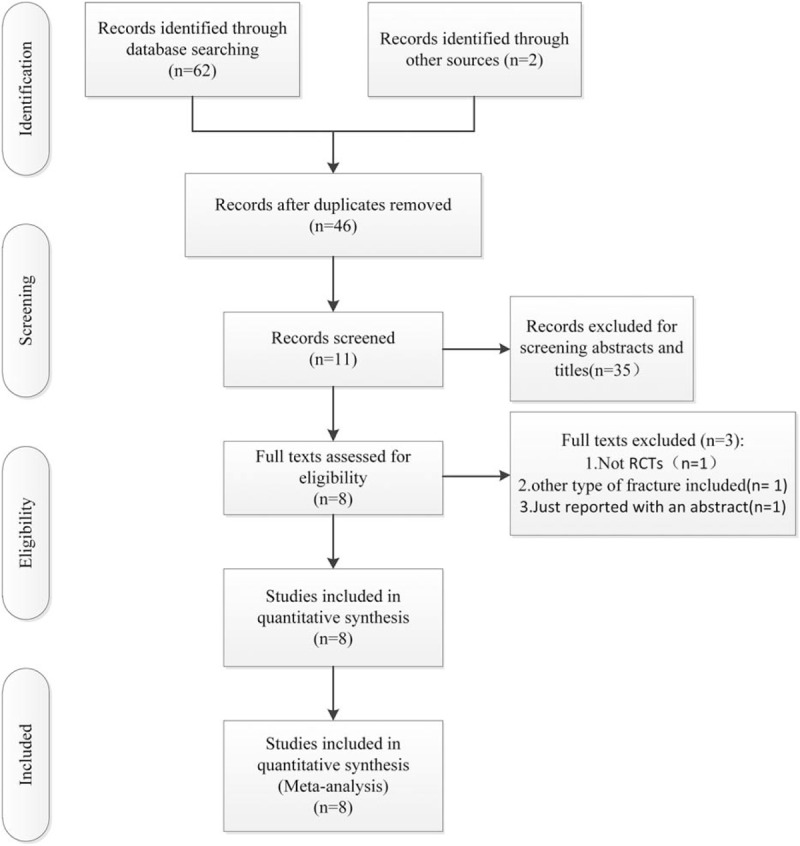
The flowchart of literature screening.

Electronic databases were searched before December 2016 by 2 independent reviewers, including Cochrane Library, EMBASE, PubMed, Web of Science, the Chinese Biomedical Literature database, and the China National Knowledge Infrastructure databases. We also checked the references of the included literatures for potentially relevant studies. There were no language restrictions. The keywords used included “randomized controlled trials,” “tranexamic acid,” and “hip fracture.” They were combined with Boolean operators. The search results are shown in Fig. 1.

### 2.2 Inclusion and exclusion criteria

Trials could be eligible for inclusion if they met the following criteria: RCTs involved the comparison of the efficacy and safety of IV TXA use in hip fracture patients; and studies included at least one of the outcome measures. Studies were excluded if: not RCTs; studies with other types of fractures included; studies with incomplete information; and duplicate publication.

### 2.3 Data extraction

Two investigators scanned the studies to extract data independently using a predefined data extraction form. The following data were collected: first author names, published year, sample size, mean age, anesthesia methods, fracture type, surgical management, intervention, control, thromboprophylaxis, transfusion criteria, and follow-up. Disagreement was resolved by consulting the reviewer. The outcomes of current meta-analysis were: total blood loss, hidden blood loss, postoperative hemoglobin decline, transfusion rates, thrombotic events, and operative time.

### 2.4 Assessment of methodological quality

Two investigators independently assessed the quality of the RCTs according to the methods of the 12-item scale.^[16]^ Each item was scored “Yes,” “Unclear,” or “No.” If a trial with a score of more than 7 “Yes” (including 7) was considered high quality, more than 4 but no more than 7 was considered moderate quality, and no more than 4 was considered low quality. Agreement on the outcome was assessed by the means of a kappa test. Any different opinions were resolved by a third reviewer.

### 2.5 Data analysis and statistical methods

The meta-analysis was conducted with STATA V.12.0 software. For continuous outcomes, the weighted mean difference (WMD) with 95% confidence interval (CI) was used. For dichotomous data, the risk difference (RD) with 95% CI was calculated as the summary statistics. Statistical heterogeneity was assessed using the value of *P* and *I*^2^. If *P* >.1 and *I*^2^ <50%, the fixed-effect model was used; otherwise, the random-effect model was used to for analysis. The assessment of publication bias and meta-regression could not be conducted, because there were just 8 studies included in our meta-analysis, and tests for them are generally performed only when at least 10 studies are involved. But subgroup analyses were conducted on mean age, hip fracture type, and surgical management. The studies which did not met the requirements of subgroup analysis were excluded. If necessary, sensitivity analysis was conducted to identify the origins of the significant heterogeneity. The Grading of Recommendations Assessment, Development, and Evaluation (GRADE) approach was used to determine the quality of each outcome.^[17]^

## 3 Results

### 3.1 Search result

A total of 64 potentially relevant references were founded. After the process of finding duplicates, 18 studies were excluded. By scanning the titles and abstracts, 35 studies were excluded from analysis. After full texts carefully read for eligibility, 3 studies were excluded, 1 was not a RCT,^[18]^ 1 was involved with other type of fracture,^[19]^ and another was with incomplete information.^[20]^ Finally, the data of 8 RCTs^[18–25]^ were pooled to make current meta-analysis. The characteristics of all included RCTs are shown in Table 1.

**Table 1:**
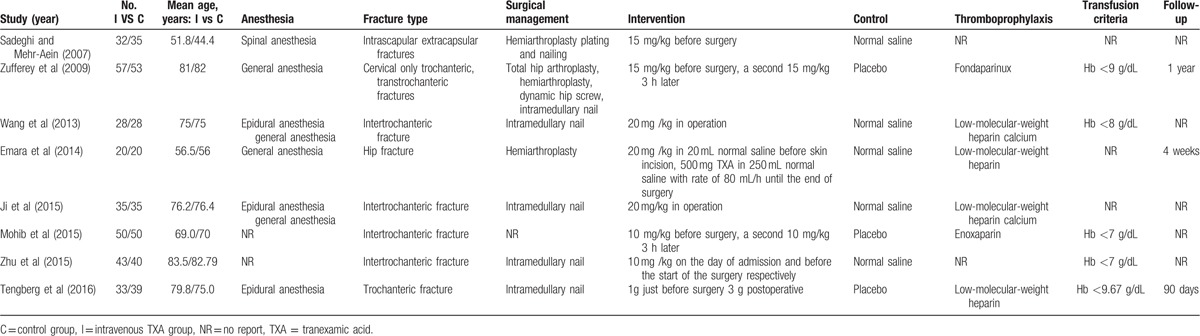
The characteristics of each study.

### 3.2 Study quality

The quality of them is shown in Table 2. The value of weighted kappa for the agreement on these studies between reviewers was excellent (Κ = 0.71). Six studies were of high quality^[18–22,25]^ and 2 studies^[23,24]^ were of moderate quality. The randomization methods were explicitly introduced in 6 studies.^[19–23,25]^ Randomization allocation was concealed adequately in 3 studies.^[19,21,22]^ Five RCTs^[18–22]^ provided the information of double blinding. None of them reported a binding of outcome assessment. But all of included studies were reported with complete outcome data.

**Table 2:**
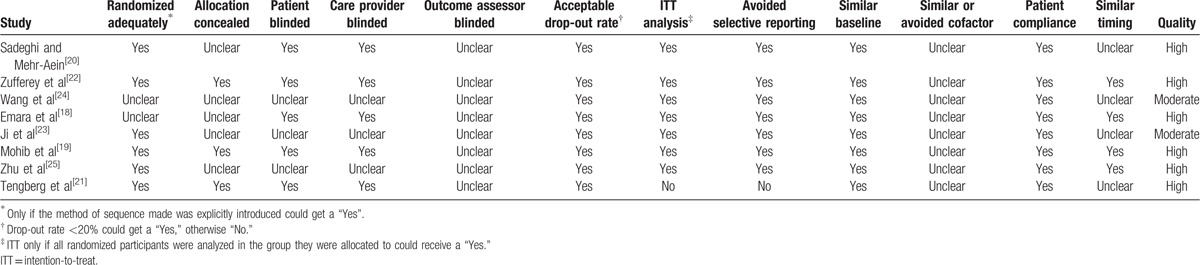
Study quality.

### 3.3 Total blood loss(ml)

**Figure 2:**
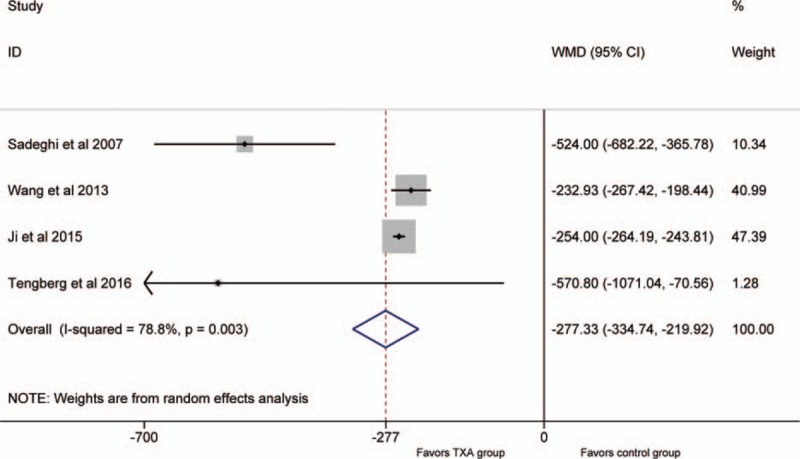
Forest plot for the total blood loss.

Four studies^[20,21,23,24]^ compared the total blood loss. The data of them were pooled to do analysis. There was a significant heterogeneity between the studies (*P* <.1; *I*^2^ = 78.8%); therefore, the random-effect model was used. The pooled results manifested that the IV TXA group had a significant decrease in the total blood loss (WMD = −277, 95%CI: −335 to −220, *P* = .000; Fig. 2).

### 3.4 Hidden blood loss(ml)

**Figure 3:**
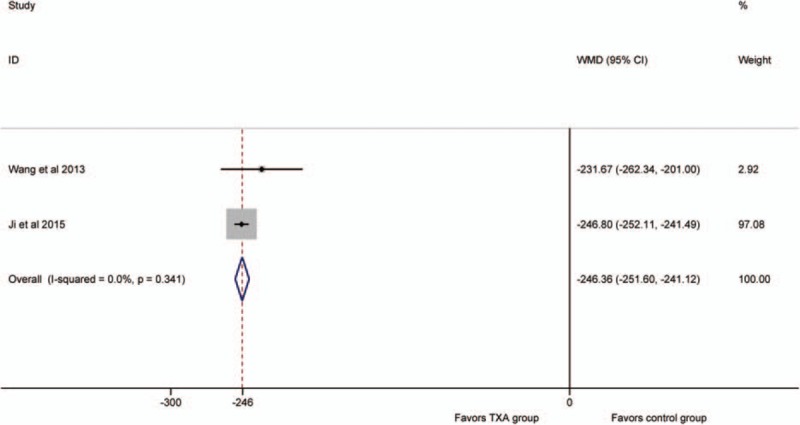
Forest plot for the hidden blood loss.

Two studies^[23,24]^ compared the hidden blood loss. So, we included them as the data of the meta-analysis. No significant heterogeneity was detected between the studies (*P* >.1; *I*^2^ = 0%); therefore, the fixed-effect model was used. The pooled results showed that the IV TXA group had a significant decrease in the hidden blood loss (WMD = −246, 95%CI: −252 to −241, *P* = .000; Fig. 3).

### 3.5 Postoperative hemoglobin decline(g/dl)

**Figure 4:**
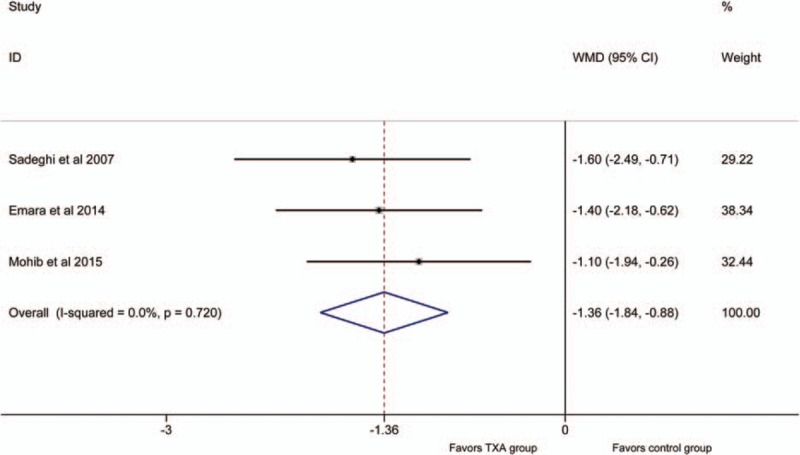
Forest plot for the postoperative hemoglobin decline.

Three articles^[18–20]^ reported the outcomes of postoperative hemoglobin decline. No significant heterogeneity was detected between the studies (*P* >.1, *I*^2^ = 0%); therefore, the fixed-effect model was used to do analysis. The pooled results demonstrated that the IV TXA groups had a lower postoperative hemoglobin decline (WMD = −1.36, 95% CI: −1.84 to −0.88, *P* = .000; Fig. 4).

### 3.6 Transfusion rate

**Figure 5:**
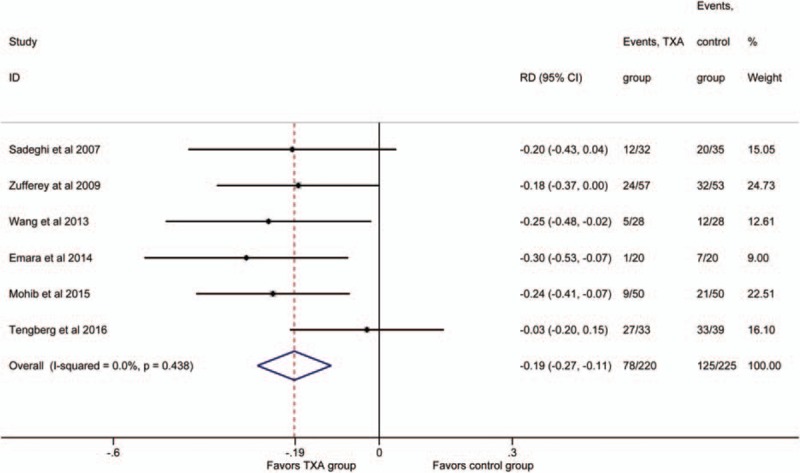
Forest plot for the transfusion rate.

Six studies^[18–22,24]^ involved the comparison of transfusion rates. No significant heterogeneity was detected between the studies (*P* >.1; *I*^2^ = 0%). Therefore, the fixed- effect model was used to do analysis. The results showed the IV TXA groups had a lower transfusion rate (RD = −0.19, 95% CI: −0.27 to −0.11, *P* = .000; Fig. 5).

### 3.7 The rate of thrombotic events

**Figure 6:**
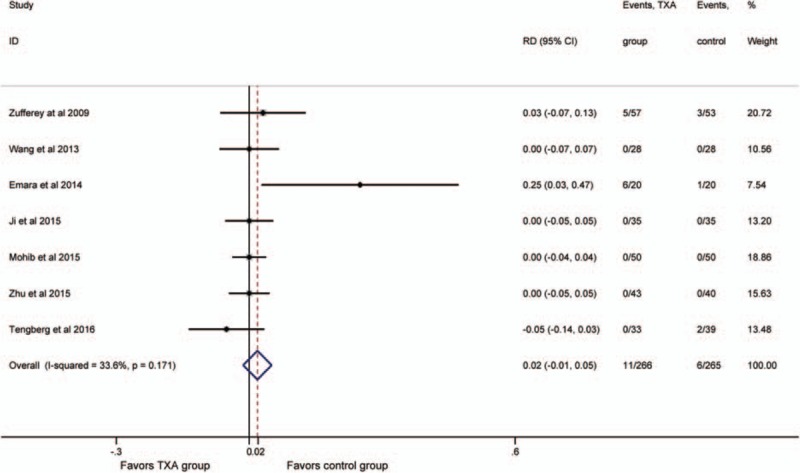
Forest plot for the rate of thrombotic events.

Seven studies^[18,19,21–25]^ reported thrombotic events. No significant heterogeneity was detected (*P* >.1; *I*^2^ = 33.6%), so the fixed-effect model was performed. It showed no significant difference in the rate of thrombotic events between the groups (RD = 0.02, 95% CI:−0.01 to 0.05, *P* = .262; Fig. 6).

### 3.8 Operative time(min)

**Figure 7:**
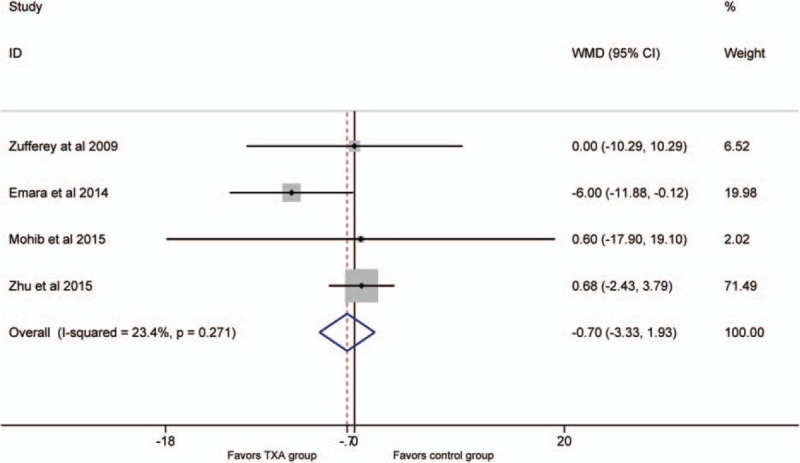
Forest plot for the operative time.

The operative time was reported in 4 studies.^[18,19,22,25]^ There was no significant heterogeneity between the 2 groups (*P* >.1; *I*^2^ = 23.4%), therefore, the fixed-effect model was used. The result manifested that no significant difference was detected (WMD = −0.7, 95% CI: −3.3 to 1.9, *P* = .6; Fig. 7).

### 3.9 Subgroup analysis

Due to a lack of related studies, subgroup analysis was just conducted in transfusion rates and thrombotic events. Concerning transfusion rates, subgroup analysis showed no significant difference regarding mean age less than 65 years (RD = −0.19; 95% CI: −0.33 to −0.04; *P* = .011) and mean age more than 65 years (RD = −0.19; 95% CI, −0.29 to −0.09; *P* = .000;), and there was no significant difference in transfusion rates with TXA identified for extracapsular hip fractures (RD = −0.18; 95% CI, −0.29 to −0.07; *P* = .001). However, transfusion rates with TXA identified for intramedullary nail fixation showed there was no statistical difference between TXA group and control group (RD = −0.13; 95% CI: −0.26 to 0.01; *P* = .076). The small size of the studies and the lack of related trials may contribute to it.

To the rate of thrombotic events, there were no significant differences in the rate of thrombotic events with TXA identified for extracapsular hip fractures (RD = −0.01; 95% CI, −0.04 to 0.02; *P* = .472) and intramedullary nail fixation (RD = −0.01; 95% CI: −0.05 to 0.02; *P* = .435)

### 3.10 Sensitivity analysis

As significant heterogeneity was detected in the outcome “total blood loss,” thus, we deleted 1 single study from the overall pooled analysis each time to check the influence of the removed dataset to the overall data. No matter which study was deleted, the outcome still kept stable. In addition, we also made sensitivity analysis on other outcomes, they were also stable. The limit data concerning IV TXA use in hip fracture surgery may contribute to the significant heterogeneity, and the difference in clinical measures should also be taken into consideration.

### 3.11 GRADE analysis

According to the results of GRADE analysis, the quality of the evidence was moderate for postoperative hemoglobin decline, the rate of complications, transfusion rates, and operative time and low for hidden blood loss and total blood loss.

## 4 Discussion

The most important finding of our meta-analysis was that systemic administration of TXA could reduce blood loss and transfusion rates without increasing thrombotic events in hip fracture surgery. These findings were similar to evidence from other meta-analysis investigating IV TXA in hip and knee arthroplasty.^[13]^ However, significant heterogeneity was detected in our outcome. The limit data concerning IV TXA use in hip fractures may contribute to it. The difference in clinical measures should also be taken into consideration.

As the elderly account for a large proportion of hip fractures, accompanying with more medical comorbidities, the patients suffering hip fractures were more susceptible to thrombotic events compared with patients undergoing elective total joint arthroplasty.^[26,27]^ Therefore, there is clinical uncertainty regarding IV TXA application in hip fracture patients. However, the potential benefits of TXA in significantly decreasing blood loss and transfusion rates following hip fracture surgery are overwhelming.^[18–25,28,29]^ In addition, TXA could bring improved functional recovery, shorter length of stay, and lower cost.^[20,26]^ Therefore, the potential benefits of TXA in hip fracture patients may outweigh the risk of TXA.

Mohib et al^[19]^ randomized 100 patients to 2 groups which demonstrated that TXA could decrease blood loss and transfusion rates without increasing rate thrombotic events during the treatment of intertrochanteric fractures. The conclusions of Wang et al,^[24]^ Ji et al,^[23]^ and Zhu et al^[25]^ were similar to them. In addition, Lee et al^[28]^ even suggested that TXA should be widely used in all patients undergoing hip hemiarthroplasty for hip fractures through a retrospectively study. However, Zufferey et al^[22]^ reported that TXA reduced the risk of erythrocyte transfusion in hip fracture surgery, but there was a trend in an increased risk of vascular events with the use of TXA. They found a 3-fold increased risk of vascular events with the use of TXA when compared with placebo (*P* = .10). Although the result was not statistically significant, it was in contrast with the previous findings of hip or knee arthroplasty where the use of TXA was without any increase in thrombotic events. Emara et al^[18]^ used thromboelastogram as a monitor for coagulation (a significant decrease in r and k and a significant increase in MA and α-angle), which favored relative hypercoagulable state in IV TXA group, and they considered that IV TXA was associated with a statistically significant increase in the incidence of thrombotic events.

Blocking the lysine-binding sites on plasminogen to inhibit activation of plasminogen and finally interfering with fibrinolysis is the mechanism of TXA. TXA itself does not increase the synthesis of fibrin, so TXA in theory does not lead to a high coagulation state after surgery, and certainly not an increase of thrombotic events. The CRASH-2 study which included 20,000 patients, most of them suffering from significant hemorrhage, reported that TXA safely reduced the risk of death in bleeding trauma patients.^[30]^

Regarding anesthetic methods, Urwin et al^[31]^ through a meta-analysis of RCTs indicated that the regional anesthetic group had a trend toward a lower incidence of myocardial infarction, confusion, and postoperative hypoxia. They made a conclusion that there were marginal advantages for regional anesthesia compared to general anesthesia for hip fracture patients in terms of early mortality and risk of deep vein thrombosis. However, a latest meta-analysis reported that types of anesthesia might not be a risk factor for hip fracture surgery.^[32]^ Due to a lack of related studies, we could not conduct a subgroup analysis to investigate it. The better anesthetic methods in hip surgery still need a further research.

The key aspects for future research: it was well established that systemic administration of TXA could reduce blood loss and transfusion rates in hip fracture surgery. The thrombotic risk is of vital importance to its recommendation; large and high-quality studies should focus on the safety of IV TXA application, especially in elderly patients. Topical TXA in hip fractures should be taken into consideration. Drakos et al^[33]^ reported that topical TXA around the fracture site in elderly patients undergoing intertrochanteric fractures surgery was safe and cost-effective, and a significant reduction in blood loss and transfused blood units, and health care cost could be achieved. In addition, Emara et al^[18]^ demonstrated that topical TXA as an effective way to decrease blood and transfusion rates was safer compared with IV TXA. A lack of systemic absorption with topical TXA may contribute to it. As the extracapsular hip fractures have a higher amount of blood loss than intrascapular hip fractures, surgeons should put more emphasis on the effects and safety of TXA in such high-risk blood loss patients. As hip fractures are frequently accompanied by a high initial blood loss, especially unstable extracapsular hip fractures, early TXA may decrease the incidence rate of preoperative anemia without increasing of thrombotic events, like the CRASH-2 study, which reported TXA successfully in hemostasis without increasing the risk of vascular events. ^[30]^ 5) Earlier surgery has been reported with a lower risk of death and lower rates of postoperative pneumonia and pressure sores among elderly patients with hip fracture.^[34]^ Early TXA may decrease the incidence rate of preoperative anemia, which may bring an earlier surgery.

Limitations identified with this study: due to a lack of clinical guideline for TXA use, the included studies showed different TXA dosage and timing still, therefore, it was impossible to make subgroup analysis regarding the dose and timing of TXA across studies. Significant heterogeneity was detected in total blood loss, the differences in fracture type, and surgical measurements and transfusion criteria may contribute to it. The GRADE analysis showed that the quality of some outcomes was low which reduced the credibility of the results. The publication bias exists.

## 5 Conclusions

It was well established that systemic administration of tranexamic acid could reduce blood loss and transfusion rates in hip fracture surgery. But the optimal regimen, dosage, and timing still need a further research. In addition, more large and high-quality randomized controlled studies are needed to focus on the safety of IV TXA application before its wide recommendation for use in hip fracture surgery.

## References

[1] Watters CL Moran WP Hip fractures—a joint effort. Orthop Nurs 2006;25:167–70. 10.1097/00006416-200605000-00003 16735846

[2] Foss NB Kehlet H Hidden blood loss after surgery for hip fracture. J Bone Joint Surg Br 2006;88:1053–9. 16877605 10.1302/0301-620X.88B8.17534

[3] Lawrence VA Silverstein JH Cornell JE Higher Hb level is associated with better early functional recovery after hip fracture repair. Transfusion 2003;43:1717–22. 14641869 10.1046/j.0041-1132.2003.00581.x

[4] Vamvakas EC Blajchman MA Transfusion-related mortality: the ongoing risks of allogeneic blood transfusion and the available strategies for their prevention. Blood 2009;113:3406–17. 19188662 10.1182/blood-2008-10-167643

[5] Newman ET Watters TS Lewis JS Impact of perioperative allogeneic and autologous blood transfusion on acute wound infection following total knee and total hip arthroplasty. J Bone Joint Surg 2014;96:279–84. 24553883 10.2106/JBJS.L.01041

[6] Carson JL Altman DG Duff A Risk of bacterial infection associated with allogeneic blood transfusion among patients undergoing hip fracture repair. Transfusion 1999;39:694–700. 10413276 10.1046/j.1537-2995.1999.39070694.x

[7] Allain JP Stramer SL Carneiro-Proietti ABF Transfusion-transmitted infectious diseases. Biologicals 2009;37:71–7. 19231236 10.1016/j.biologicals.2009.01.002

[8] Eubanks JD Antifibrinolytics in major orthopaedic surgery. J Am Acad Orthop Surg 2010;18:132–8. 20190103

[9] Strang CM Hachenberg T Current strategies to minimize of blood loss during radical prostatectomy. Anasthesiol Intensivmed Notfallmed Schmerzther 2013;48:494–9. quiz 500-1. 23929170 10.1055/s-0033-1352497

[10] Ngichabe S Obura T Stones W Intravenous tranexamic acid as an adjunct haemostat to ornipressin during open myomectomy. A randomized double blind placebo controlled trial. Ann Surg Innov Res 2015;9:1–6. 26568770 10.1186/s13022-015-0017-y PMC4644022

[11] Zabeeda D Medalion B Sverdlov M Tranexamic acid reduces bleeding and the need for blood transfusion in primary myocardial revascularization. Ann Thorac Surg 2002;74:733–8. 12238832 10.1016/s0003-4975(02)03784-0

[12] Myles PS Smith JA Forbes A Tranexamic acid in patients undergoing coronary-artery surgery. N Engl J Med 2016;376:136–48. 27774838 10.1056/NEJMoa1606424

[13] Chen Y Chen Z Cui S Topical versus systemic tranexamic acid after total knee and hip arthroplasty: A meta-analysis of randomized controlled trials. Medicine 2016;95:e4656. 27741100 10.1097/MD.0000000000004656 PMC5072927

[14] Ueno M Sonohata M Fukumori N Comparison between topical and intravenous administration of tranexamic acid in primary total hip arthroplasty. J Orthop Sci 2015;21:44–7. 26755385 10.1016/j.jos.2015.10.011

[15] North WT Mehran N Davis JJ Topical vs intravenous tranexamic acid in primary total hip arthroplasty: a double-blind, randomized controlled trial. J Arthroplasty 2015;31:1022–6. 26703193 10.1016/j.arth.2015.11.003

[16] Furlan AD Pennick V Bombardier C Updated method guidelines for systematic reviews in the Cochrane back review group. Spine 2009;34:1929–41. 19680101 10.1097/BRS.0b013e3181b1c99f

[17] Schünemann HJ Oxman AD Brozek J Grading quality of evidence and strength of recommendations for diagnostic tests and strategies. BMJ 2008;336:1106–10. 18483053 10.1136/bmj.39500.677199.AE PMC2386626

[18] Emara WM Moez KK Elkhouly AH Topical versus intravenous tranexamic acid as a blood conservation intervention for reduction of post-operative bleeding in hemiarthroplasty. Anesth Essays Res 2014;8:48–53. 25886103 10.4103/0259-1162.128908 PMC4173581

[19] Mohib Y Rashid RH Ali M Does tranexamic acid reduce blood transfusion following surgery for inter-trochanteric fracture? A randomized control trial. J Pak Med Assoc 2015;65:S17–20. 26878513

[20] Sadeghi M Mehr-Aein A Does a single bolus dose of tranexamic acid reduce blood loss and transfusion requirements during hip fracture surgery? A prospective randomized double blind study in 67 patients. Acta Medica Iranica 2007;45:437–42.

[21] Tengberg PT Foss NB Palm H Tranexamic acid reduces blood loss in patients with extracapsular fractures of the hip: results of a randomised controlled trial. Bone Joint J 2016;98B:747–53. 10.1302/0301-620X.98B6.36645 27235515

[22] Zufferey PJ Miquet M Quenet S Tranexamic acid in hip fracture surgery: a randomized controlled trial. Br J Anaesth 2010;104:23–30. 19926634 10.1093/bja/aep314

[23] Ji ZW Xia L Yao LD Effect of tranexamic acid on perioperative hidden blood loss in aged patients receiving intramedullary fixation for treatment of intertrochanteric fractures. Chin J Gerontol 2015;7:1853–4.

[24] Wang XD Jiang YY Wang JZ Clinical research of tranexamic acid on the hidden blood Ioss after the treatment of intertrochanteric fractures with proximal femoral nail anti-rotation (PFNA). Med Res Educ 2013;30:51–4.

[25] Zhu YS Jiang C Li J Effect of tranexamic acid on perioperative hidden blood loss in aged patients receiving intramedullary fixation for treatment of intertrochanteric fractures. J Trad Chin Orthop Trauma 2015;27:16–8.

[26] Gausden EB Garner MR Warner SJ Tranexamic acid in hip fracture patients: a protocol for a randomised, placebo controlled trial on the efficacy of tranexamic acid in reducing blood loss in hip fracture patients. BMJ Open 2016;6:e010676. 10.1136/bmjopen-2015-010676 PMC4916621 27329438

[27] Simmons J Sikorski RA Pittet JF Tranexamic acid: from trauma to routine perioperative use. Curr Opin Anaesthesiol 2015;28:191–200. 25635366 10.1097/ACO.0000000000000165 PMC4408016

[28] Lee C Freeman R Edmondson M The efficacy of tranexamic acid in hip hemiarthroplasty surgery: an observational cohort study. Injury 2015;46:1978–82. 26190627 10.1016/j.injury.2015.06.039

[29] Vijay BS Bedi V Mitra S Role of tranexamic acid in reducing postoperative blood loss and transfusion requirement in patients undergoing hip and femoral surgeries. Saudi J Anaesth 2013;7:29–32. 23717228 10.4103/1658-354X.109803 PMC3657919

[30] Collaborators CT Shakur H Roberts I Effects of tranexamic acid on death, vascular occlusive events, and blood transfusion in trauma patients with significant haemorrhage (CRASH-2): a randomised, placebo-controlled trial. West Indian Med J 2010;59:612–24. 21702233

[31] Urwin SC Parker MJ Griffiths R General versus regional anaesthesia for hip fracture surgery: a meta-analysis of randomized trials. Br J Anaesth 2000;84:450–5. 10823094 10.1093/oxfordjournals.bja.a013468

[32] Zuo D Jin C Shan M A comparison of general versus regional anesthesia for hip fracture surgery: a meta-analysis. Int J Clin Exp Med 2016;8:20295–301. PMC4723788 26884943

[33] Drakos A Raoulis V Karatzios K Efficacy of local administration of tranexamic acid for blood salvage in patients undergoing intertrochanteric fracture surgery. J Orthop Trauma 2016;30:409–14. 26978136 10.1097/BOT.0000000000000577

[34] Simunovic N Devereaux PJ Sprague S Effect of early surgery after hip fracture on mortality and complications: systematic review and meta-analysis. CMAJ 2010;182:1609–16. 20837683 10.1503/cmaj.092220 PMC2952007

